# Acupuncture for the Treatment of Pain – A Mega-Placebo?

**DOI:** 10.3389/fnins.2019.01110

**Published:** 2019-10-17

**Authors:** Frauke Musial

**Affiliations:** Department of Community Medicine, National Research Center in Complementary and Alternative Medicine, NAFKAM, Faculty of Health Science, UiT – The Arctic University of Norway, Tromsø, Norway

**Keywords:** acupuncture, sham acupuncture, placebo needles, pain, placebo, expectation, pain matrix

## Abstract

Several control conditions, such as penetrating sham acupuncture and non-penetrating placebo needles, have been used in clinical trials on acupuncture effects in chronic pain syndromes. All these control conditions are surprisingly effective with regard to their analgesic properties. These findings have fostered a discussion as to whether acupuncture is merely a placebo. Meta-analyses on the clinical effectiveness of placebo revealed that placebo interventions in general have minor, clinically important effects. Only in trials on pain and nausea, including acupuncture studies, did placebo effects vary from negligible to clinically important. At the same time, individual patient meta-analyses confirm that acupuncture is effective for the treatment of chronic pain, including small but statistically significant differences between acupuncture and sham acupuncture. All acupuncture control conditions induce *de qi*, a distinct stimulation associated with pain and needling which has been shown to be a nociceptive/pain stimulus. Acupuncture therefore probably activates the pain matrix in the brain in a bottom-up fashion via the spino-thalamic tract. Central nervous system effects of acupuncture can be modulated through expectations, which are believed to be a central component of the placebo response. However, further investigation is required to determine how strong the influence of placebo on the attenuation of activity in the pain matrix really is. A meta-analysis of individual participant functional magnetic imaging data reveals only weak effects of placebo on the activity of the pain network. The clinical acupuncture setting is comprised of a combination of a distinct neurophysiological stimulus, the needling stimulus/experience, and a complex treatment situation. A broader definition of placebo, such as that proposed by Howick (2017) acknowledges a role for expectation, treatment context, emotions, learning, and other contextual variables of a treatment situation. The inclusion of particular treatment feature as a definitional element permits a contextual definition of placebo, which in turn can be helpful in constructing future clinical trials on acupuncture.

## The Effects of Acupuncture and Placebo in Clinical Pain Studies

### Clinical Acupuncture Studies on Chronic Pain Conditions

In clinical acupuncture studies on pain, acupuncture control conditions (often called sham acupuncture) show strong clinical effects. The three most frequently applied acupuncture control conditions are: (i) points that differ from the acupuncture points are needled; (ii) true acupuncture points are needled, but only very gently, and with very thin needles (minimal acupuncture) and (iii) so-called placebo needles that do not penetrate the skin are used (for further discussion see paragraph “Needling associated procedures as sham acupuncture conditions in clinical trials”).

In many of these clinical pain studies on acupuncture, acupuncture control conditions are more effective than standard medical care. In the German Acupuncture Randomized Trials (ART) studies, for example, in which true acupuncture was compared with minimal acupuncture and waiting list control, both true and minimal acupuncture were superior to being on a waiting list for the diagnosis of migraine, tension headache, or chronic back pain (Linde et al., 2005; Melchart et al., 2005; Brinkhaus et al., 2006). The only exception was knee arthritis, where only true acupuncture brought significant relief (Witt et al., 2005).

The German Acupuncture trials (GERAC studies) followed a slightly different design by comparing true acupuncture with minimal-acupuncture and standard medical care (Molsberger et al., 2006a, b). The results were similar: The treatment effect of acupuncture or minimal acupuncture was significantly superior to standard medical care and the effect was strong with no significant differences between the acupuncture conditions (Haake et al., 2007). The close similarity between true acupuncture, sham- and minimal acupuncture has raised the question as to whether the actual procedure of acupuncture is a placebo. Nonetheless, one of the consequences of these trials, which were funded by German health insurance companies, was that acupuncture is now recognized for by many German health insurance companies as a viable treatment for several conditions.

One of the most central questions in clinical studies is, however, whether and how the treatment which is to be tested compares to the available gold standard. A systematic review (Linde et al., 2009) consisting of twenty-two trials with a total of 4,419 patients came to the conclusion that acupuncture is not only at least as effective for migraine prophylaxis as prophylactic drug treatment but that it also has less side effects. Once again, no difference was established between true acupuncture and sham acupuncture procedures. In a systematic review on acupuncture treatment for tension type headache (Linde et al., 2009), the trials included were more heterogeneous (11 trials with 2,317 patients) with regard to the control conditions while the results were less homogeneous. The authors nonetheless came to the conclusion that acupuncture constitutes a valuable non-pharmacological treatment alternative.

Unlike the results from the large clinical acupuncture studies on pain syndromes, several brain-imaging studies in defined patient populations revealed relatively clear therapeutic acupuncture effects. For example, acupuncture therapeutic brain imaging studies in neuropathic pain (carpal tunnel syndrome) (Maeda et al., 2013, 2017) showed distinct true acupuncture-related neuroplasticity in the somatosensory cortex. This neuroplasticity is specifically related to increased neural functionality in the periphery (median nerve function) as well as to symptom improvement. One particularity of these two studies may be partly due to the use of electro acupuncture which constitutes a somewhat powerful acupuncture treatment.

In conclusion, the fact that sham acupuncture was equally effective as true acupuncture in almost all of the clinical trials on acupuncture has fostered a discussion as to whether acupuncture constitutes a particularly effective placebo, or whether sham acupuncture is an inadequate control.

### How Powerful Is Placebo? Meta-Analyses on Clinical Placebo Effects

At the same time, the placebo effect, and in particular its potential clinical relevance was under discussion. While basic scientists claimed that a strong placebo effect existed (Benedetti et al., 2005), the authors of the first major Cochrane review on the topic (Hrobjartsson and Gotzsche, 2003), concluded that there was no evidence to suggest that placebo interventions have clinically important effects in general. Moreover, in the only clinical condition in which a significant placebo effect could be detected, i.e., pain, the result was restricted to subjective continuous outcomes, and could not be clearly distinguished from bias (Hrobjartsson and Gotzsche, 2003). The authors came to the same conclusion after incorporating new evidence and trials (Hrobjartsson and Gotzsche, 2004a,b). Despite extending the search to more clinical conditions, the conclusion that placebo interventions in general have no important clinical effects still prevailed (Hrobjartsson and Gotzsche, 2010).

However, on the basis of the new data material, the authors qualified their conclusion as follows: “in certain settings placebo interventions can influence patient-reported outcomes, especially pain and nausea, though it is difficult to distinguish patient-reported effects of placebo from biased reporting. The effect on pain varied, even among trials with low risk of bias, from negligible to clinically important. Variations in the effect of placebo were partly explained by variations in how trials were conducted and how patients were informed” (Hrobjartsson and Gotzsche, 2010). It is important to bear in mind that, since these meta-analyses contained acupuncture trials, in particular with regard to pain, they included and calculated sham- acupuncture conditions as placebo interventions. As discussed later in more detail, non-pharmacological placebos such as sham acupuncture as well as sham surgery maybe clinically more effective than oral pharmacological placebos (Meissner et al., 2013). This has certainly been the case in trials on migraine prophylaxis using network meta-analysis. The interpretation that the effect of non-pharmacological placebos is similar to pharmacological placebos may, therefore, be misleading and may undermine the effectiveness of the verum condition, here true acupuncture.

Consequently, the discussion on the relevance of placebo in general – and its role for acupuncture in particular – was intense and at times heated (Hrobjartsson and Gotzsche, 2006). Nonetheless, even a meta-analysis on acupuncture treatment for pain which included a systematic review of randomized clinical trials with true acupuncture, sham acupuncture, and no acupuncture groups did not reach any conclusion (Madsen et al., 2009). The aim was to explore the analgesic effect of acupuncture and sham acupuncture and to ascertain whether the type of the placebo acupuncture applied is associated with the estimated effect of acupuncture. Only three-armed randomized clinical trials consisting of a true acupuncture, a sham acupuncture (or other similar controls), and a non-acupuncture group were included. However, this did not alter the results and conclusion: “A small analgesic effect of acupuncture was found, which seems to lack clinical relevance and cannot be clearly distinguished from bias. Whether needling at acupuncture points, or at any site, reduces pain independently of the psychological impact of the treatment ritual is unclear” (Madsen et al., 2009). The general conclusion from meta-analytic approaches to placebo is therefore that placebo effects are small, usually not clinically significant, and that the acupuncture effects in pain trials are inconsistent, dependent on study design and outcome, and very probably the result of bias.

### Sham Acupuncture: A Particularly Powerful Placebo?

While several meta-analyses on acupuncture pain trials did not differ, a meta-analyses technique combining individual patient data (Vickers et al., 2010) came to a completely different conclusion. The first individual patient data meta-analyses, using data from 29 of 31 eligible RCTs, with a total of 17,922 patients, established that acupuncture is effective for the treatment of chronic pain (Vickers et al., 2012). Furthermore, they noted small, but statistically significant differences between true- and sham acupuncture. The authors state that “these differences are relatively modest, suggesting that factors in addition to the specific effects of needling are important contributors to the therapeutic effects of acupuncture.” An update confirms the results and conclusion (Vickers et al., 2017) and a further detailed analysis of the data set suggests that the clinical benefit persists over time (MacPherson et al., 2017).

The interpretation that acupuncture in general is a particularly powerful placebo is predominantly due to the strong effects of sham acupuncture conditions as control conditions in clinical trials. Nonetheless, with regard to the question as to the clinical relevance of these effects, the comparison of true acupuncture/sham acupuncture conditions to other control conditions, in particular no-treatment controls or routine care controls, is relevant.

In their meta-analysis over 37 trials involving 5,754 patients Linde et al. (2010a) concluded that sham acupuncture conditions have much greater non-specific effects than placebo interventions. This meta-analysis also focused on three-armed trials, including non-acupuncture control groups. Unlike Madsen et al. (2009) the authors distinguished sham acupuncture conditions from inert placebo conditions in the discussion and raised the question as to whether sham acupuncture conditions differ from other placebos in that the former induce particularly large unspecific effects. Such an interpretation would have substantial methodological consequences for the design of clinical trials. The authors also raised the issue as to how such a finding should be evaluated in the light of the clinical relevance of sham acupuncture (Linde et al., 2010a, b). The same issue was raised by Moffet (2009) who, in his systematic review, also deemed sham acupuncture conditions to be as clinically relevant as true acupuncture conditions.

To answer the question as to whether sham acupuncture interventions are generally more clinically effective than other placebos (Linde et al., 2010b), reanalyzed the data from the Cochrane review of Hrobjartsson and Gotzsche (2010) on placebo effects in all clinical conditions. They concluded, with all due caution given the heterogeneity of the included trials, that sham acupuncture conditions are in fact associated with stronger non-specific effects than other physical and pharmacological placebos. As already mentioned above, the phenomenon that sham acupuncture conditions are more clinically effective than, for example, oral placebos, was confirmed by Meissner et al. (2013) in their elegant systematic review on the differential effectiveness of different placebo treatments in trials on migraine prophylaxis. This network meta-analysis revealed that both sham acupuncture and sham-surgery were clinically more effective than oral pharmacological placebos.

Penetrating sham acupuncture has been criticized on account of the fact that it may actually constitute a less effective form of acupuncture treatment. Therefore, non-penetrating devices which mimic the acupuncture procedure but which do not actually penetrate the skin have been developed (Streitberger and Kleinhenz, 1998; Park et al., 2002). Nonetheless, a systematic review by Zhang et al. (2015) reveals precisely the same effect for non-penetrating needles as for other sham acupuncture procedures. They conclude that neither the Streitberger nor the Park device is an inert control intervention. Moreover, sixteen studies recorded adverse events in true and sham acupuncture alike.

Nonetheless, while focusing on the issue of sham acupuncture conditions, it should be borne in mind that acupuncture has also been shown to be clinically more effective than for example non-steroidal anti-inflammatory drugs (NASIDs). In a study on chronic back pain patients, Toroski et al. (2018) ascertained that electro-acupuncture treatment was clinically effective and, moreover, more cost effective than treatment with non-steroidal anti-inflammatory drugs.

In summary, it is generally accepted that acupuncture is clinically effective in treating chronic pain states. There is a significant, but rather small difference between acupuncture and sham acupuncture. At the same time, sham acupuncture conditions appear to be more effective than other physical and pharmacological placebos.

## Needling-Associated Procedures Such as Sham Acupuncture Conditions in Clinical Trials

Three frequently employed control strategies are generally defined as sham acupuncture. Two of these methods penetrate the skin: (i) other points than acupuncture points, are needled. This type of control procedure is used to investigate the site (or point)-specific effects of acupuncture (Moore and McQuay, 2005); (ii) applying minimal acupuncture; true acupuncture points are needled, but only very gently and with extremely thin needles. This procedure, which is used for mode-specific control of acupuncture effects, should enable the physician to define the appropriate “dose” of peripheral sensory stimulation (Backer et al., 2002). A combination of these two strategies is sometimes used. A third procedure utilizes devices with non-penetrating devices similar to the so-called “Streitberger needle” or the “Park-Sham” device. These procedures are similar in that they have the appearance of an acupuncture needle but do not penetrate the skin (Streitberger and Kleinhenz, 1998; Park et al., 2002; Chae et al., 2018). These devices are often called “placebo needles.”

On the basis of the findings that sham acupuncture conditions appear to be more effective than other physical and pharmacological placebos, the issue has been raised as to whether sham acupuncture is physiologically active and, as such, not acceptable as a placebo control (Birch, 2006; Langevin et al., 2006, 2011; Lundeberg et al., 2008, 2011; Lund et al., 2009), Moreover, the choice of appropriate control conditions in clinical acupuncture trials on chronic pain syndromes poses a particular challenge, given that it is not possible to blind the acupuncturist. Double blinding is therefore essentially impossible which, in turn, substantially increases the risk of bias. In their recent review Chae et al. (2018) not only discuss the problems associated with non-penetrating sham acupuncture devices but also focus on the methodological challenges associated with trial design while introducing possible solutions with their benefits and shortcomings.

Several of the physiological mechanisms identified by Chae et al. (2018) are liable to account for some of the effects of placebo needles and cover the physiological effects of penetrating sham acupuncture: tactile stimulation is believed to be specific to the intervention and divided into the sensory discriminative and affective social component of the procedure. Some of the particularities of all forms of acupuncture/sham acupuncture/minimal acupuncture is the impossibility of blinding, possibly resulting in greater expectation-related placebo effects than when using a placebo pill. Nonetheless, expectation is also a neurophysiological effect which originates in the brain and which thus exerts its effect top-down.

## Possible Neurobiological Mechanisms of Acupuncture and Sham Acupuncture

### C-Fiber Touch as Potential Mediator for the Affective-Social Dimension of Acupuncture

C-fiber touch (CT’s) are tactile low threshold mechanosensitive fibers which are sensitive to gentle touch and which form a second touch system, with pleasant touch as a modality. They are assumed to represent the positive hedonic aspects of gentle touch (McGlone et al., 2007, 2014; Abraira and Ginty, 2013; Musial and Weiss, 2014). Thanks to animal experiments, this system has been known for quite some time and its role for humans has already been acknowledged. In humans, this system has been proposed to represent the neurobiological substrate, inducing feelings of calm and well-being related to gentle touch (McGlone et al., 2007, 2014; Abraira and Ginty, 2013; Musial and Weiss, 2014).

The particular role of touch for humans is not new to us, even though the particular C-fiber touch system has not yet been fully described for humans. For example, holding hands reduces the neural response to threat in an fMRI paradigm, even if the person holding the hand is a complete stranger (Coan et al., 2006). As Chae et al. (2018) point out, touch is a central factor in many medical procedures and an integral factor in all acupuncture conditions, regardless of whether or not the device penetrates the skin.

The touch component *per se* therefore constitutes a distinct neurobiological pathway, uncommon in all non-touch placebos such as a placebo pill. Moreover, it is represented by a specific pathway where the origin of stimulation is generated in the periphery of the body and travels in a bottom-up fashion to the brain. From here, it exerts a complex affective-emotional reaction (McGlone et al., 2007, 2014; Abraira and Ginty, 2013; Musial and Weiss, 2014). At the same time, this pathway is common to all acupuncture control conditions, thereby threatening the validity of virtually all of these conditions (Campbell, 2006; Lund et al., 2009).

### *De qi* and the Sensory-Discriminative Aspects of Acupuncture

Inserting a needle and penetrating the skin is a stimulus of vital importance. Such a procedure will stimulate pain pathways and signal information of an injury, and is therefore recognized by the brain as a potential threat to the body’s integrity. It therefore comes as no surprise that acupuncture procedures induce the strong and distinct activation of pain-related areas in the brain (Zhao, 2008; Chae et al., 2013). According to Zhao “Acupuncture analgesia is manifested only when the intricate feeling (soreness, numbness, heaviness, and distension) of acupuncture in patients occurs following acupuncture manipulation” (Zhao, 2008). This sensation, known as *de qi*, is believed to be fundamental to the therapeutic outcome of acupuncture (Choi et al., 2013; Chae et al., 2018).

To date, several studies have shown that even non-penetrating placebo needle procedures induce *de qi* sensations that are indistinguishable from the effect of real acupuncture needles (Chae, 2017; Chae et al., 2018). Taken together, these findings indicate that even non-penetrating acupuncture placebos are capable of stimulating sensory-discriminative pathways, including pain sensation. The current evidence therefore suggests that acupuncture – or indeed any other kind of needling procedure – constitutes a nociceptive and/or pain signal: Acupuncture (e.g., ST 36) activates pain-related brain structures (Biella et al., 2001; Pariente et al., 2005; Beissner et al., 2012; Theysohn et al., 2014); for overview and discussion see Wang et al. (2008), Zhao (2008), and the activation is dependent on the needling sensation (Beissner et al., 2012). Moreover, a meta-analysis across 28 fMRI studies of acupuncture needling showed an activation of the pain matrix (sensorimotor cortical network, including the insula, thalamus, and anterior cingulate cortex (ACC), as well as both the primary and secondary somatosensory cortices) (Chae et al., 2013). In summary, there is ample evidence that acupuncture *per se* constitutes a nociceptive/pain stimulus which influences brain networks in a bottom-up fashion.

Under the premise that the therapeutic acupuncture effects are at least partly mediated through the pain pathway of the sensory-discriminative system, the processing of nociceptive signals in the spinal cord must be taken into account to explain the effect of needling and its related *de qi* sensation. A needling stimulus in a painful situation, be it clinically manifested such as in a pain syndrome or experimentally induced in the laboratory, is liable to excite two mechanisms on the level of the spinal cord: segmental (gate-control) (Mayer et al., 2000; Irnich and Beyer, 2002; Stux et al., 2003) and spino-medullary (diffuse noxious inhibitory control, DNIC) (Le Bars et al., 1989; Le Bars, 2002; Le Bars and Willer, 2002, 2007; Le Bars and Cadden, 2007) processes.

While gate control is, neurophysiologically speaking, somewhat short-lived (Fields et al., 2005), DNIC is a strong long-term effect, inasmuch as it persists for several minutes after its induction. Such a response is typical of neuroplastic changes (Le Bars and Willer, 2002, 2007). DNIC characterizes an intrinsic mechanism for inhibiting pain that becomes activated when an additional nociceptive stimulus is applied to a region far removed (heterotopic) from the initial region receiving nociceptive stimulation (Le Bars et al., 1989; Le Bars, 2002; Le Bars and Willer, 2002; Le Bars and Cadden, 2007; Le Bars and Willer, 2007). This phenomenon is often classified under the superordinate concept of “counter irritation” (Le Bars et al., 1989) and is mediated via a spino-medullary loop (Le Bars, 2002; Le Bars and Willer, 2007).

In humans, experimental paradigms which test the effect of additional pain stimuli on an already existing experimental pain stimulus, and which are thus homologous to DNIC are often called “heterotopic noxious conditioning stimulations (HNCS)” (Sprenger et al., 2011) or “conditioned pain modulation (CPM)” (Bjorkedal and Flaten, 2012). Sprenger et al. (2011) used a tonic cold pressor task and phasic additional pain stimulation in an fMRI paradigm and noticed a clear HNCS effect with marked endogenous analgesia. This outcome was accompanied by reduced activity in classical pain-related brain structures and the recruitment of an opiate-dependent, descending pain control system (naloxone blockade). In women Bjorkedal and Flaten (2012) were able to show that a CPM response can be reduced by expectations.

Acupuncture has been shown to exhibit rather strong effects in an HCNS/CPM design (Choi et al., 2011a, b; Musial et al., 2012). Moreover, acupuncture decreased somatosensory-evoked potential amplitude to noxious stimuli in anesthetized volunteers in an HCNS/CPM design (Meissner et al., 2004), thus showing clear acupuncture effects in unconscious humans. This is an interesting observation, given that all cortical effects such as expectations, suggestions, beliefs etc, can be excluded as confounding factors in subjects under deep anesthesia. Further evidence from fMRI studies shows less brain activity in somatosensory areas in response to pain following acupuncture stimulation (modulation of cold pain: (Zhang et al., 2003) and electrical pain: (Theysohn et al., 2014).

In conclusion, acupuncture therapeutic effects are seen to be related to the *de qi* sensation, which is characterized as a pain-related sensation. There is evidence that acupuncture and related conditions act via a pain signal elicited in the periphery and traveling bottom-up to the brain. These peripheral nociceptive signals may undergo modulation via spinal and spino-medullary mechanisms. Since virtually all placebo/sham acupuncture procedures are known to induce *de qi* ensations that are indistinguishable from real acupuncture, it should be borne in mind that this pain-related, somatosensory-discriminative mechanism akin to acupuncture is common to all true acupuncture/sham acupuncture/minimal acupuncture, and placebo acupuncture conditions.

### Enhanced Expectation as a Mediator for the Strong Effects of “Sham”- and Placebo Acupuncture Conditions?

Despite various creative approaches such as the above discussed non-penetrating placebo needles (Streitberger and Kleinhenz, 1998; Park et al., 2002; Chae et al., 2018), an adequate experimental control of acupuncture for the treatment of chronic pain is scarcely possible, particularly since the acupuncturist cannot be blinded. One of the characteristics of all acupuncture and related procedures is, therefore, the impossibility of blinding, which can lead to greater expectation-related placebo effects than with other placebos (for overview and discussion see Chae (2017), Chae et al. (2018).

Expectation is a key mediator of the placebo response (Kirsch, 1999; Enck et al., 2008; Colloca and Miller, 2011a,b) and its relevance for the clinical effectiveness has already been well documented [(Moffet, 2009; Linde et al., 2010a; Chae et al., 2018)]. Interestingly enough, while it is widely accepted within placebo research that enhancing the placebo effect during drug treatment is desirable (Enck et al., 2013; Evers et al., 2018), the possibility that expectation – and thus placebo – may play a role in clinical acupuncture effects has been held against acupuncture as a treatment, in particular in public discourse. The argument is that acupuncture may merely represent a “placebo” without so-called “specific effects.” This is contradictory in view of the fact that, physiologically speaking, the expectancy-mediated placebo effect *per se* has meanwhile been reasonably well defined and represents a rather distinct neurophysiological mechanism (Enck et al., 2008; Wager and Atlas, 2015; Geuter et al., 2017; Schafer et al., 2018).

The individual placebo response has been under close scrutiny ever since the 1990s (Benedetti et al., 1995; Benedetti, 1996; Amanzio and Benedetti, 1999; Benedetti et al., 2005). In an experimental pain-tolerance setting, Benedetti et al., showed how conditioning and expectation processes both play a role in placebo analgesia. The expectation-induced placebo response can be completely blocked by the opioid antagonist naloxone (Benedetti, 1996), whereas the conditioning-dependent placebo response can be blocked by naloxone only when conditioning was carried out with an opioid (Amanzio and Benedetti, 1999). Thus, expectation-dependent placebo responses are, in principle, mediated by the endogenous opioid system, whereas conditioning-dependent placebo effects depend on the particular subsystem that had been conditioned. The opioid-dependent mechanism of expectation-induced placebo analgesia is also supported by neurophysiological imaging studies in man, showing that an opioid-dependent cortical network becomes activated during a placebo response (Petrovic et al., 2002; Zubieta et al., 2005). Acupuncture analgesia is similar to the expectation-dependent placebo effect in that it too can be blocked by naloxone, i.e., it is also opioid-dependent [for an overview, see (Mayer et al., 2000)].

## Acupuncture, Placebo, and the Pain Matrix

Data from imaging studies have confirmed that not only expectation but also learning and context factors play a role in placebo effects [for overview and discussion see Enck et al. (2008), Wager and Atlas (2015), Geuter et al. (2017), Schafer et al. (2018)]. Moreover, context and social factors are welcome modulators for expectation and associative learning. Most of these models are based on results from pain studies.

With regard to the placebo response, the ventromedial prefrontal cortex (vmPFC), the dorsolateral prefrontal cortex (dlPFC), the lateral orbitofrontal cortex (lOFC), nucleus accumbens, insula, amygdala, hypothalamus, and periaqueductal gray are generally regarded as crucial (Wager and Atlas, 2015; Geuter et al., 2017; Schafer et al., 2018). All these areas increase activity in response to placebo manipulations related to pain stimulation (Wager and Atlas, 2015), albeit the periaqueductal gray (PAG) and the dorsal anterior cingulate cortex (dACC) respond in a more differentiated fashion, and appear to have a modulatory function within the placebo network (Wager and Atlas, 2015). The vmPFC seemingly plays a particularly essential role in the mediation of expectation (Geuter et al., 2017). Thus, the vmPFC represents the part of the placebo brain network that connects situational factors to neurophysiological responses (Geuter et al., 2017).

At the same time, the placebo network appears to be independent from the pain network, even though some structures, primarily the PAG and the dACC, seem to have a modulatory function in both networks (Wager and Atlas, 2015; Geuter et al., 2017). This conceptualization fits well with a number of experimental findings, indicating that the perception of pain is also dependent on the context (Keltner et al., 2006). In one fMRI study, for instance (Koyama et al., 2005) were able to show that, to a great extent, the expectation of one particular stimulus intensity determined its cortical representation, regardless of whether or not it was anticipated to be painful. As the magnitude of the expected pain increased, activation increased simultaneously in the thalamus, insula, prefrontal cortex, and ACC. Pain-intensity-related brain activation was identified in a widely distributed set of brain regions and overlapped partially with expectation-related activation in other regions, including the anterior insula and ACC (Koyama et al., 2005).

Thus, the evidence available with regard to placebo effects on pain in experimental human and animal studies suggests that placebo effects are mediated by a network of brain structures that differ from the pain network. However, the placebo network influences and modulates the pain network, relying on several factors such as context, learning and prior experiences, instructions, etc. These factors probably influence the placebo network by way of expectations. Expectations are fundamental inasmuch as they may signal reward (e.g., reduced pain). Anticipated reward will activate the reward system in the brain, a system that involves the nucleus accumbens, the amygdala, the ventral tegmental area, and the orbitofrontal cortex (Enck et al., 2008). The neurotransmitters of this system are dopamine and opioid peptides, and this network is usually investigated with regard to drug dependencies and reinforcement. In summary, instructions, suggestions, and other types of social information can have powerful effects on pain and these changes are mediated by top-down control via the involvement of prefrontal regions, particularly the dorsolateral and ventromedial prefrontal cortex (Koban et al., 2017).

As already elucidated, current evidence suggests that any kind of needling procedure constitutes a nociceptive and/or pain signal (Biella et al., 2001; Pariente et al., 2005; Beissner et al., 2012; Theysohn et al., 2014); for overview and discussion see Wang et al. (2008), Zhao (2008), Beissner et al. (2012), and that acupuncture needling (a meta-analysis across 28 fMRI studies) induces an activation of the pain matrix (sensorimotor cortical network, including the insula, thalamus, anterior cingulate cortex, and both primary and secondary somatosensory cortices) (Chae et al., 2013). Moreover, data from healthy humans subjects in response to needling of commonly used acupuncture points for the treatment of pain syndromes (manual acupuncture at LI4, ST36) revealed a partial overlap with the pain matrix as well as a deactivation of a limbic-paralimbic-neocortical network (Hui et al., 2009; Claunch et al., 2012). This limbic deactivation is correlated to the psychophysical response modulating the bottom-up nociceptive signal. It therefore modulates brain networks in response to acupuncture stimulation. Assuming that acupuncture is a pain stimulus that travels bottom-up and activates the pain networks, it must also be assumed that expectations, instructions, emotions, and other context factors modulate acupuncture effects.

In an elegant study Lee et al. (2015) modulated and manipulated an acupuncture stimulus by instruction. One group was told that the needling was part of an acupuncture treatment, whereas the other group was informed about the needle insertion only, and that this might be a painful intervention. In addition to their acupuncture stimulation, all participants received the same sensory stimuli which included a pain stimulus. The brain activation in response to the pain stimulus in parts of the pain network was significantly lower in those participants who had been informed that the needle insertion was part of an acupuncture treatment. At the same time, the needling within a therapeutic context activated reward circuits in the brain and modulated the pain matrix.

The results from brain imaging studies emanating from placebo research as well as the results of Chae et al. (2013) and Lee et al. (2015) suggest that: (i) the pain matrix and the placebo network are primarily independent brain networks; (ii) the pain matrix and the placebo network interact and the placebo-induced modulation of the pain-matrix is mediated by expectation; (iii) acupuncture activates the pain matrix and is a nociceptive/pain stimulus, and (iv) acupuncture as a nociceptive/pain stimulus is modulated by placebo effects, in particular expectation.

Nonetheless, how powerful the modulation of the pain-matrix through placebo effects actually is remains to be elucidated. A meta-analysis of individual participant functional magnetic resonance imaging data combined the results of 603 healthy individuals from 20 studies and investigated the effects of placebo intervention on the pain matrix (Zunhammer et al., 2018). The analysis revealed rather moderate analgesic effects on subjective pain reports and only a very small effect of the placebo condition on the pain matrix. The authors concluded that placebo treatments affect pain through brain mechanisms (top down) and that these influences are largely independent from the bottom-up effects of nociceptive processing.

## Is Acupuncture a Mega-Placebo? On the Relativity of Placebo

In particular the neurobiological developments in the understanding of the mechanisms of the placebo response have confirmed that the concept placebo encompasses a wide variety of modalities such as emotions, instructions, suggestions, and other types of social information mediated by alterations in prefrontal brain areas (Koban et al., 2017). These factors are often described as the “unspecific” effects of a particular treatment setting, indicating that the treatment effects are mainly psychological in nature and independent from the treatment provided.

To date, the concept of specificity at brain level, which is reminiscent of the historic theory of phrenology, may be questioned. Defined brain areas, such as the PAG or the dACC play a role in a number of brain functions. This fact tallies well with the concept that brain functions are performed by the activity of neural networks. The concept of neural networks also explains why the perception of pain is context-dependent (Koyama et al., 2005; Keltner et al., 2006); a finding that is relevant for the explanation of pain-related placebo effects.

Howick (2017) proposes a broader definition of placebo which goes beyond the claim that a placebo should consist of an inert “intervention simulation.” His modified version of Grünbaum’s definition of placebo emphasizes the role of expectations, and thus includes emotions, learning, and other contextual variables. Moreover, his definition assigns a special role to the peculiarities of a treatment situation, including special treatment features, and thus partly allows for a contextual definition of placebo. However, it is worth bearing in mind that Grünbaum’s original article (Grunbaum, 1986), which relates to placebo effects in psychiatry and psychotherapy, remains somewhat controversial (Enck and Zipfel, 2019). To what extent this concept can be applied to other non-pharmacological treatments such as acupuncture – which is in many ways more “physical” than psychotherapy – may not be entirely consensual in the field of placebo research.

Nonetheless, Howick’s interpretation of Grünbaum’s definition (Grunbaum, 1986) provides an opening for the role of treatment theory and thus calls for a broadening of the definition of placebo beyond the role of expectation. In his paper, Howick (2017) uses the acupuncture/sham acupuncture/placebo needle discussion as a case study to illustrate that treatment theory is fundamental to the definition of a placebo condition and that it does not suffice to control exclusively for expectation. In his example, point specificity as treatment theory will lead to an acupuncture-related placebo concept which interprets needling at non-acupuncture points as “placebogenic” [page 1371, Howick (2017)]. In his case study on acupuncture, he argues that independent evidence supports the assumption that the placebo needle itself (Streitberger needle) cannot be a true placebo. He pinpoints that the core challenge in the interpretation of the clinically effective control procedures for the acupuncture treatment of pain states is the lack of an accepted therapeutic theory for acupuncture.

Howick’s broadened concept of placebo not only allows for the defined use of placebo controls beyond the pharmacological context but also includes also non-pharmacological interventions. Nonetheless, as he demonstrates in his acupuncture case study, his definition demands and includes a defined therapeutic theory. With regard to many interventions out of the spectrum of complementary and alternative medicine, this may still constitute a challenge, such as in the case of acupuncture. Further clinical studies addressing treatment theory of acupuncture as well as the placebo/expectation issue are therefore required.

The field of placebo research has accepted that placebo might not be as distinctively defined as it is necessary for conducting a clinical trial in the non-pharmacological arena. Placebo effects are viewed as positive and useful factors of a treatment, particularly in clinical practice, and are considered to be a part of *every* regular treatment (Evers et al., 2018). With this definition, the debate as to what extent a treatment is dependent on placebo becomes less pungent and the focus is relocated to the most important question, namely to: what helps patients, what relieves their pain, and how can they be treated most effectively. This pragmatic approach aims to maximize all the positive factors of a treatment that can be recruited while minimizing risk and negative effects, including nocebo (Evers et al., 2018).

## Author Contributions

The author confirms being the sole contributor of this work and has approved it for publication.

## Conflict of Interest

The author declares that the research was conducted in the absence of any commercial or financial relationships that could be construed as a potential conflict of interest.
